# Supplementary material for: Astragalus Shiitake—A Novel Functional Food with High Polysaccharide Content and Anti-Proliferative Activity in a Colorectal Carcinoma Cell Line
Source: Nutrients. 2022 Jun 2;14(11):2333. doi: 10.3390/nu14112333 (PMC9182587; doi:10.3390/nu14112333)
Supplement: Supplementary file 1 [file nutrients-14-02333-s001.zip › nutrients-1641819-supplementary.pdf]

Figure S1

Comparing the effect of old reported UEA extraction technique [32] and new UEA extraction technique (developed in this study) on the total yield and antiproliferative activity of HAS-B polysaccharides.

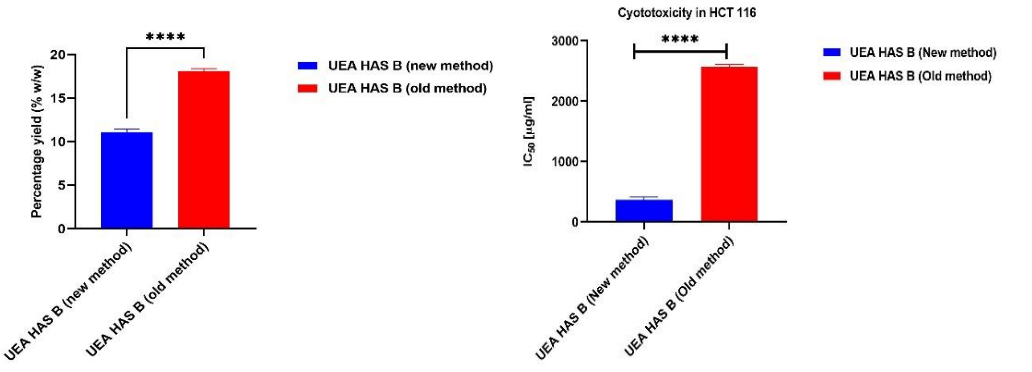

(A1) (A2)

Figure S1. Effect of old and new UEA extraction techniques on total polysaccharide yield and cytotoxic activity against HCT 116. A1) Old extraction technique had significant increase in polysaccharideyield ($P < 0.0001$, indicated as ****) compared to new extraction technique applied in this study. A2) New extraction technique developed in this study showed significantly high cytotoxicity activity ($P < 0.0001$, indicated as ****) compared to the old reported technique.

Figure S2: Comparison of the yield and cytotoxicity of UEA HAS-B polysaccharides extracted from two different enzyme mixtures. ACPVP is the mixture of four different enzymes; Alcalase, Cellulase, Pectinase and Vinotaste-pro in a ratio 1:1:1:1 and ACP is the mixture of three different enzymes; Alcalase, Cellulase and Pectinase in a ratio 1:1:1.

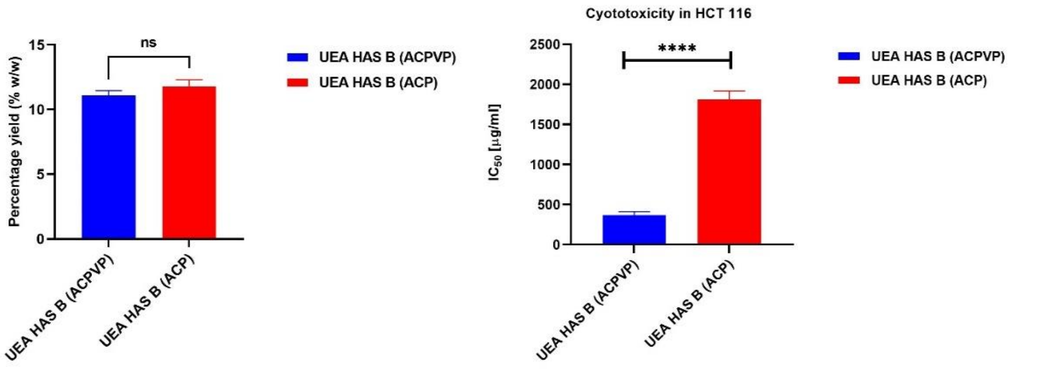

(B1) (B2)

Figure S2. Effect of enzyme mixtures on yield and antiproliferative activity of HAS-B polysaccharides. B1) The percentage yield of UEA HAS-B polysaccharides, extracted from new enzyme mixture ACPVP (developed in this study) was not significantly different (indicated as "ns") compared to previously reported enzyme mixture ACP. B2) UEA HAS-B polysaccharides from new enzyme mixture ACPVP showed significant increase in cytotoxic activity against HCT 116 ($P < 0.0001$, indicated as ****) compared to ACP.
